# Design and Implementation of Poultry Farming Information Management System Based on Cloud Database

**DOI:** 10.3390/ani11030900

**Published:** 2021-03-22

**Authors:** Haikun Zheng, Tiemin Zhang, Cheng Fang, Jiayuan Zeng, Xiuli Yang

**Affiliations:** 1 College of Engineering, South China Agricultural University, 483 Wushan Road, Guangzhou 510642, China; haikun0619@gmail.com (H.Z.); gu5457111@gmail.com (C.F.); zsural@126.com (J.Z.); xlyscau@scau.edu.cn (X.Y.); 2 National Engineering Research Center for Breeding Swine Industry, Guangzhou 510642, China; 3 Guangdong Laboratory for Lingnan Modern Agriculture, Guangzhou 510642, China

**Keywords:** poultry farming, information management, cloud database, disease detection

## Abstract

**Simple Summary:**

Informatization can effectively improve the production and management efficiency in the poultry farming process. In this study, a management system was designed to realize the acquisition, transmission, storage, and management of information, and upload the data to the cloud database to increase the flexibility and scalability of the system. On the basis of realizing production management functions, the system also incorporates an office management module, thus forming a complete data chain in production activities, so as to conduct farming data mining and accurate traceability in the next stage of the work. In particular, the system also adds poultry disease detection module supports to achieve the purpose of healthy farming. The research provides an information management plan for the intensive poultry farming model, and the designed management system may be the starting point of a future intelligent poultry farming management system based on cloud services and big data technology.

**Abstract:**

Aiming at breaking down the bottleneck problems of different scale of poultry farms, the low profitability of poultry farming, and backward information management in China, a safe and efficient information management system for poultry farming was designed. This system consists of (1) a management system application layer, (2) a data service layer, and (3) an information sensing layer. The information sensing layer obtains and uploads production and farming information through the wireless sensor network built in the poultry house. The use of a cloud database as an information storage carrier in the data service layer eliminates the complex status of deploying local server clusters, and it improves the flexibility and scalability of the system. The management system application layer contains many sub-function modules including poultry disease detection functions to realize the visual management of farming information and health farming; each module operates independently and cooperates with each other to form a set of information management system for poultry farming with wide functional coverage, high service efficiency, safety, and convenience. The system prototype has been tested for the performance of wireless sensor network and cloud database, and the results show that the prototype is capable of acquiring and managing poultry farming information.

## 1. Introduction

Modern poultry farming companies need a complete management system to assist companies in managing their daily production activities. The system should cover such things as personnel office management, purchase–sales–inventory management, environmental monitoring and control in poultry houses, and monitoring of individual poultry information. At the same time, it also needs to include traceability management of products, diagnosis, and early warning of poultry diseases to meet the need for future development.

With the development of large-scale and intensive poultry farming, more intelligent and automated technologies and methods have been applied in poultry farming [1,2], such as radio frequency identification technology [3], Internet of Things technology (IoT), and cloud technology [4]. At the same time, there are methods such as poultry disease detection, poultry diet monitoring [5], environmental monitoring in poultry houses [6,7], product tracking and traceability, and abnormal detection in poultry houses to achieve precision farming. Yu Ligen et al. developed a network-based data acquisition system using LabVIEW software for environmental monitoring in poultry management [8], which describes the construction of data acquisition system hardware and the process of data acquisition. The method also provides a reference for us to build an environmental monitoring module. British Irvine explored the British broiler meat value chain [9] and provided a method for constructing the traceability module in the poultry farming management system through its in-depth analysis of the value chain. Research on applying wireless sensing systems along with mobile networking and cloud platform to some agricultural systems in crops [10] has provided us with new ideas to develop a similar information system for poultry farming.

In recent years, more and more researchers have devoted themselves to the study of precision poultry farming [11,12]. Some researchers help farmers control and monitor the health status of poultry through IOT, imagery analytics, and other technologies [13]. Other researchers build online platforms and using smart sensors to record and manage production information in real time [14,15,16]. Although wireless sensing and cloud platform techniques are well advanced, there is no complete system that covers all of the functions to meet needs for poultry farming management. The technical difficulties include the unified construction of the system, the reasonable division of functional modules, the good mutual cooperation between modules, the interaction of software and hardware, and the intelligentization of the system.

This paper reports the conceptual design of a poultry farming information management system with a cloud database as the core hub, through the connection of the underlying hardware facilities in the poultry house and the upper management system and the cloud database to manage the daily office work and production management tasks of poultry farming enterprises. In addition, this cloud-based management system also pays more attention to the storage and management of data information by separating the database system from the software system. The ultimate goal of the poultry farming information management system is to expand the development of the poultry industry management system with big data analysis capability.

## 2. Overall System Architecture

**Figure 1:**
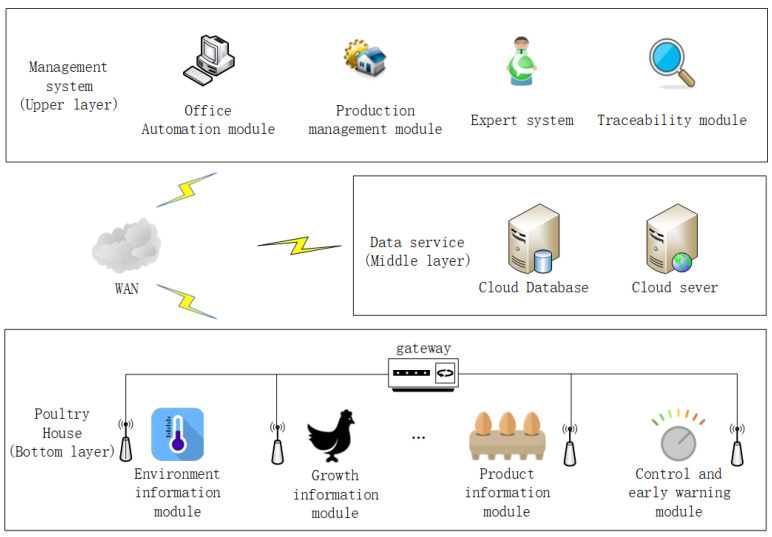
Overall structure of the system.

Figure 1 shows the overall structure of the system. The system is divided into three layers, which are the upper management system, the intermediate data service layer (also known as the middle layer), and the underlying layer (also known as the bottom layer, including hardware facilities in the poultry house).

The upper layer is a software management system that provides a good visual interface. The management system is divided into an office automation module, a production management module, an expert system, and a traceability module. As for the middle layer, the cloud database is used to store the data and information generated by the upper layer and the bottom layer, and at the same time construct a reasonable network environment to solve mutual communication problems by configuring the underlying server. At the bottom layer, in poultry house(s), environmental sensors, Wi-Fi receiving and transmitting devices, and single-chip microcomputers can be configured to timely acquire and transmit environmental information and poultry individual information (including poultry weight information, feed intake data information, drinking water data information, poultry egg quality information, etc.). Ventilation fans, evaporative cooling pads, heaters, and other equipment placed in the poultry house to regulate environmental parameters such as temperature and humidity in the house.

### 2.1. System Network Construction and Transmission Method

An environmental parameter information sensor (including temperature and humidity sensor, ammonia sensor, carbon dioxide sensor, hydrogen sulfide sensor, light intensity sensor, etc.), feed intake data monitoring module, drinking water data monitoring module, video monitoring system, fans, evaporative cooling pads, heaters, feeding, and manure cleaning facilities in the poultry house together form the local area network system in the house. This section mainly reports the information transmission methods, local transmission strategies, and configuration of network nodes in poultry houses.

Data transmission between the poultry house and the house environmental control system is primarily provided by a suitable wired communication system, such as a fieldbus. There are some disadvantages (e.g., configure too many network endpoints, device address assignment rules, and other issues) of using a full wireless system. Therefore, the local area network in the house uses a wireless/wireline hybrid construction [17]. As shown in Table 1, among the three commonly used wireless transmission modes (i.e., Bluetooth, Wi-Fi, and ZigBee), the Wi-Fi technology has the longest transmission distance and the fastest transmission speed [18]; therefore, Wi-Fi technology was selected as the wireless transmission method in the poultry house selects.

### 2.2. Cloud Database

The Alibaba Cloud Database RDS service was used in the system, as it has a good visual operation interface and numerous auxiliary analysis tools. It can generate database-related files such as E-R diagrams (Entity Relationship Diagram) and data dictionary with one click, and it can also generate test data to ensure the test works during database development. The database uses a relational database, and the database version is MySQL5.7. As the core hub of a poultry farming information management system, the cloud database should carry out requirements analysis, concept design, logical structure design, construction of the E-R model, design table structure, and primary-foreign key relationships in the process of design and construction.

The intranet address of the system can be accessed by the Alibaba Cloud Server, which has the advantages of fast reading speed and convenient setup. The external network address can be accessed by Internet users with access rights, and the database can be read and written. In the design stage, the selected database memory is 1024 MB, 1 core CPU, the storage capacity is 20 GB, and the maximum number of connections is 2000. It is confirmed in the actual development test that this configuration can meet the development needs.

### 2.3. Upper Management System

The management system uses the C++ language as the main development tool, the latest Qt5 framework as an open source support library, and the Qt Creator as an IDE (Integrated Development Environment) for compilation and development.

**Figure 2:**
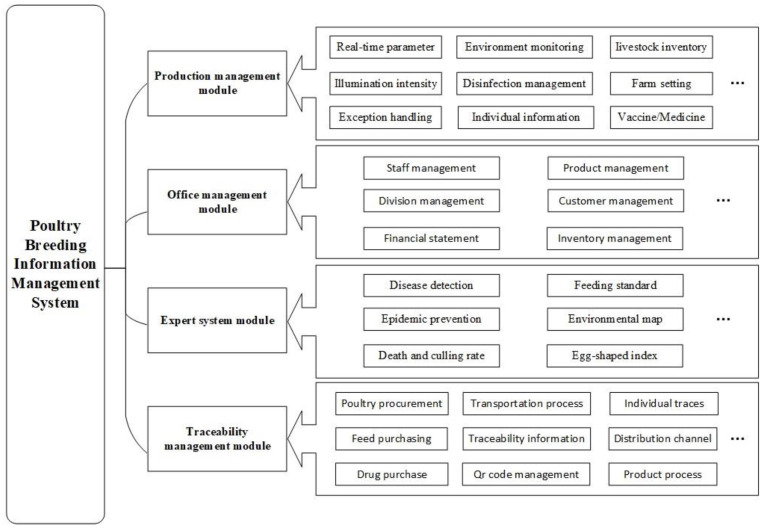
Functional framework of poultry farming information management system.

Figure 2 shows the functional framework of the poultry farming management system. The whole system is divided into four functional modules. The production management module mainly realizes the monitoring of environmental parameters in the poultry house, the monitoring of the growth information of individual poultry, and the management of the production operations in the poultry house. The office management module mainly fulfills the business tasks such as personnel management, financial management, and invoicing. The expert system module combines artificial intelligent technology such as data mining and machine learning to (1) realize egg shape index analysis; (2) provide feeding standards, breeding recommendations, mortality analysis, and other functions, and (3) realize poultry disease diagnosis and an early warning system based on audio and image analysis of poultry. Modules are functionally independent, with data-sharing capability.

## 3. Wireless Sensor Network Design

The bottom layer of the system is mainly composed of wireless sensor networks, which are used to manage terminal nodes and the uploaded data information.

**Figure 3:**
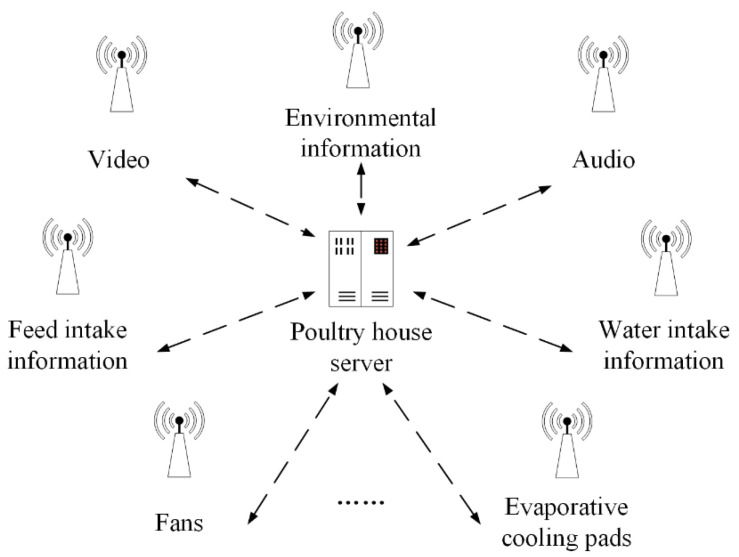
The composition of the wireless sensor network in the poultry house.

The composition of the wireless sensor network in the poultry house is shown in Figure 3. Each terminal node uses AT commands to automatically search for the wireless network by name and join it. After joining the network, it can independently obtain the device IP address and server IP address. Open the transparent transmission mode through the AT command, and use the UDP transmission protocol to transmit the data information. At the same time, in order to summarize and forward the data information uploaded by each terminal node, a data server should also be configured in the wireless sensor network in the poultry house.

The wireless sensor network and various types of intelligent equipment can solve the problem of obtaining and transmitting various kinds of rearing information (e.g., environmental information, poultry weight information, poultry dietary information). This section mainly takes environmental information as an example to introduce the implementation process of information acquisition and upload. In the example, four environmental information sensing units are deployed in the wireless sensor network.

### 3.1. Poultry House Server

A data server should be configured in the wireless sensor network in the poultry house to process and forward the data information obtained by the terminal nodes in the network. Therefore, the server in the poultry house should choose a controller that has a processor, operating system, wireless network card, and can store programs. Considering the harsh working environment in the poultry house, this research selected the Industrial Personal Computer (IPC) with stronger waterproof, dustproof, and anti-interference capabilities than the data server.

In addition to the attributes and characteristics of ordinary computers, it also has stronger anti-interference ability and long-term uninterrupted working ability, which are suitable for use in the context of poultry farming environment. This research work selects an industrial control computer as the server in the poultry house to process and upload the data generated by the terminal node of the wireless sensor network, and the performance parameters are shown in Table 2.

In order to realize the processing and uploading of the data information generated by the terminal node, and at the same time realize the management and control of the wireless sensor network in the poultry house, a set of server programs is designed and loaded on the server in the poultry house to achieve the above-mentioned purpose.

### 3.2. Environmental Information Sensing Unit (EISU)

**Figure 4:**
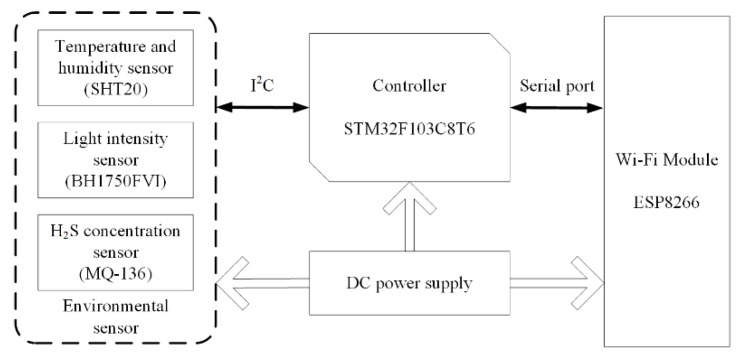
The structure diagram of the environmental information sensing unit structure.

As the terminal node of the wireless sensor network, the environmental information sensing unit (EISU) in the poultry house should contain various digital environmental sensors, such as temperature and humidity sensors, carbon dioxide concentration sensors, hydrogen sulfide concentration sensors, light intensity sensors, wind speed sensors, etc., and it should also be equipped with a micro processor and wireless transmission module. The structure diagram is shown in Figure 4, and the performance parameters of the environmental sensors used are shown in Table 3, which are provided by the manufacturer.

**Figure 5:**
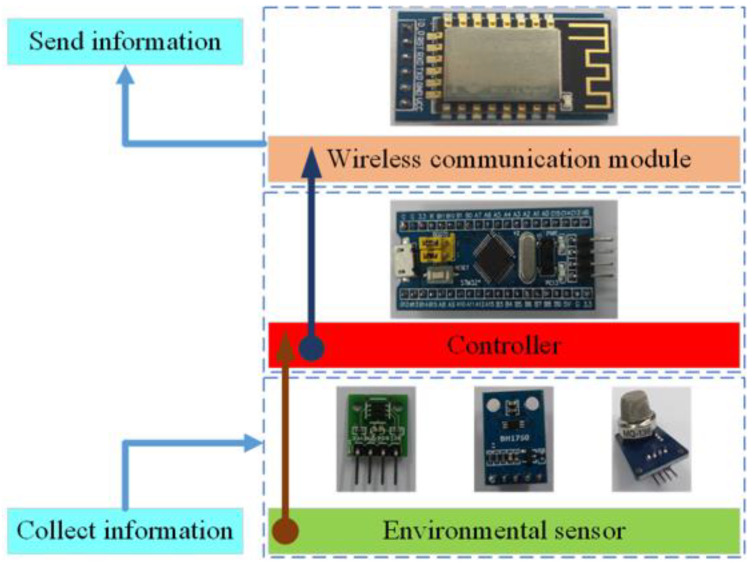
Flow chart of multi-threaded processing environment information data program.

Various environmental sensors are used to sense and measure the parameter values of the surrounding environment and using the I2C protocol to transmit them to the microcontroller through the data bus. The microcontroller is responsible for packaging the environmental data information according to the data packet format in Table 4 and uploading it to the poultry house server through the wireless transmission module; the diagram of the data flow is shown in Figure 5. Finally, the poultry house server uploads the data to the cloud database.

### 3.3. Data Collection and Transmission

During the data transmission process of the wireless sensor network in the poultry house, there is no need to establish a one-to-one connection, only a one-to-many connection is needed to realize the communication between the terminal node and the server. Due to the large amount of data information transferred between the terminal node and the server and the number of transmissions, combined with the actual functional requirements, the network communication between the terminal node and the server of the wireless sensor network in the poultry house mainly uses UDP transmission protocol and socket technology.

**Figure 6:**
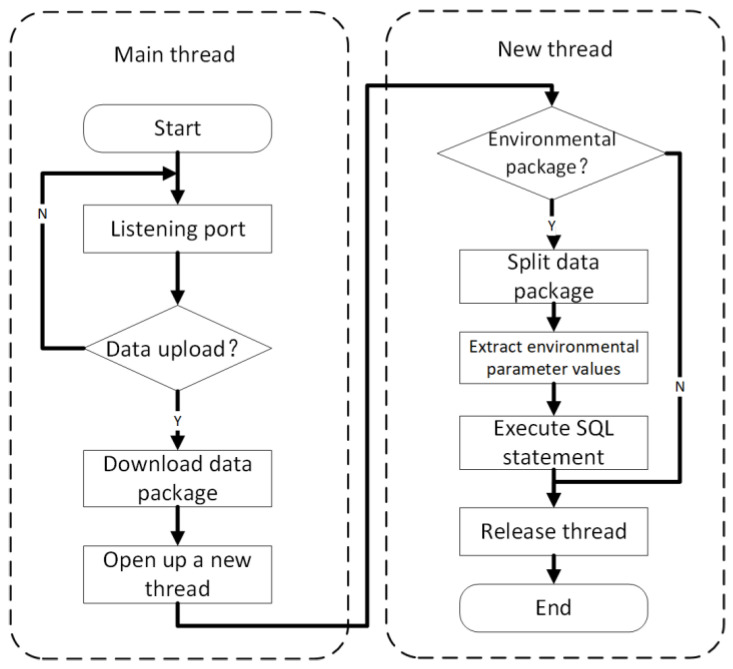
Data flow diagram of environmental information sensing unit.

The terminal node packages the data in a certain format and uses socket technology to send messages to the specific IP address and port number of the poultry house server. When the data information is successfully sent to the server in the poultry house, the server program downloads the data packet from the monitored port; then, it uses the specified transmission format to split the information, and it executes the corresponding SQL statement according to the type of data to upload the data to the cloud database; the data processing flow chart is shown in Figure 6.

**Figure 7:**
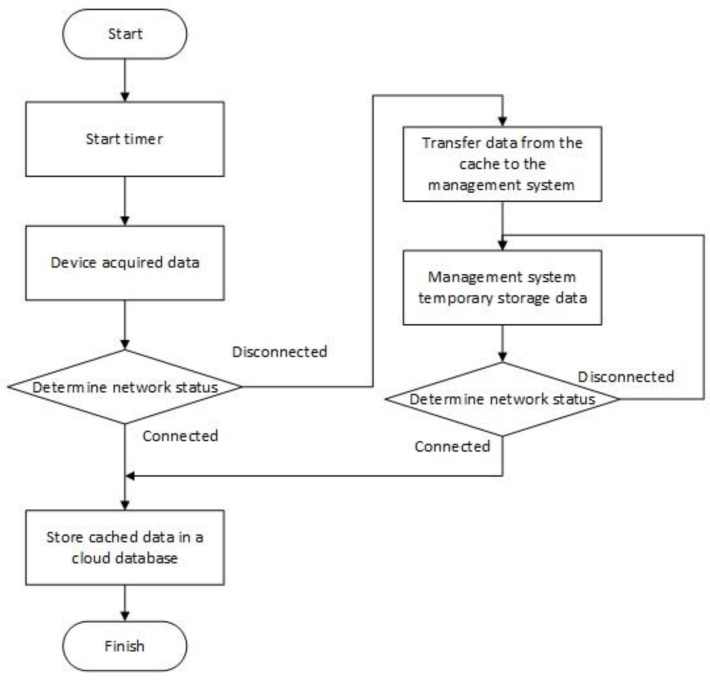
Local transmission strategy of the routing node flow chart.

Since there are many data transmission devices placed in the poultry house, it is necessary to set a routing node in the poultry house to forward the network data package to the cloud database. Taking into account the problem of network fluctuations, the server in the house may be disconnected from the cloud database, and the data information generated in the poultry house has real-time and continuous characteristics, so a data protection program must be designed to prevent data loss information. Figure 7 shows the local transmission strategy of the routing node, taking into account the network factors such that the system data security is improved.

## 4. Management System Implementation

**Figure 8:**
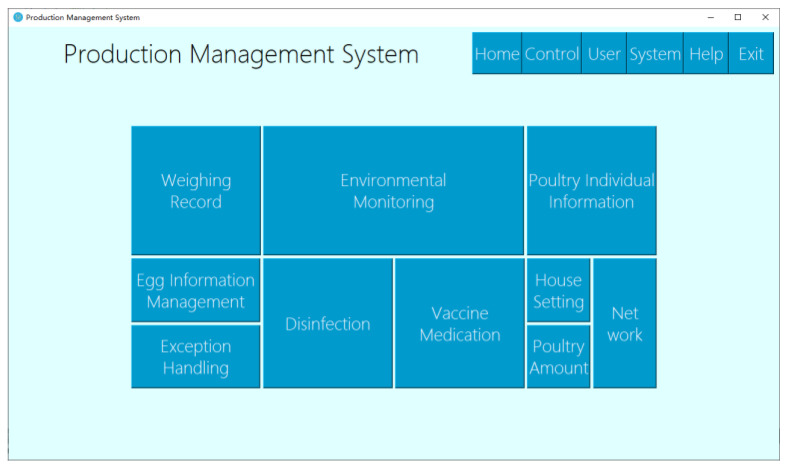
Management system home, includes ten function modules: weighing record, environmental monitoring, poultry individual information, egg information management, exception handing, disinfection, vaccine medication, house setting, poultry amount, and network.

The process of constructing the poultry farming information management system could be modularized, constructed, and tested one by one. First, a comprehensive platform for the management system can be built, and then the functional modules can be assembled. Figure 8 illustrates the home page of the management system.

This section mainly introduces the design and implementation effects of the environmental information management module and the poultry disease monitoring module.

### 4.1. Environmental Information Management Module

This module mainly realizes real-time monitoring of environmental information in the poultry house, and it can display the fluctuations of different environmental parameters. It can provide early warning in time when the environment in the house is abnormal and at the same time carry out good storage and management of historical environmental data.

**Figure 9:**
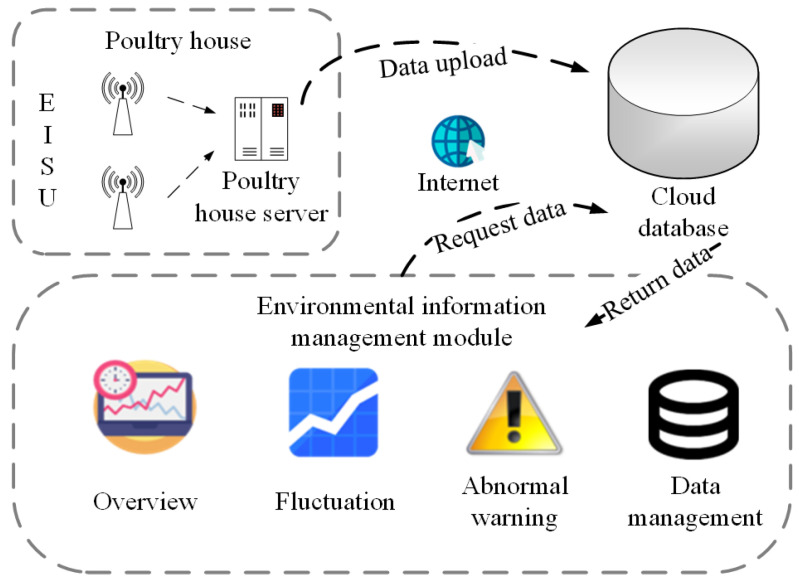
The business process chart of environmental information management module. EISU means environmental information sensing unit.

**Figure 10:**
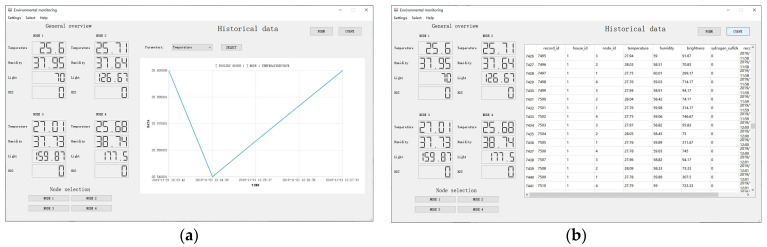
The software interface effect of an environmental information management module, (**a**) fluctuating presentation of environmental data, (**b**) historical environmental data, including temperature, humidity, light, H_2_S concentration, ammonia concentration, carbon dioxide concentration, etc.

The business process diagram of this module is shown in Figure 9. The EISUs upload the collected environmental information to the cloud database through the poultry house server. The cloud database is responsible for storing and managing the information. The environmental information management module makes active requests, queries the cloud database, and then display the feedback data through this module, so as to realize the management function of environmental information in the poultry house. The software interface effect of this module is shown in Figure 10.

### 4.2. Poultry Disease Monitoring Module

The disease monitoring and early warning function of this module is based on a large number of research results of our laboratory team, mainly through the analysis of poultry video and audio information to obtain information about the health status of poultry or related information about poultry disease [19,20,21,22,23,24].

**Figure 11:**
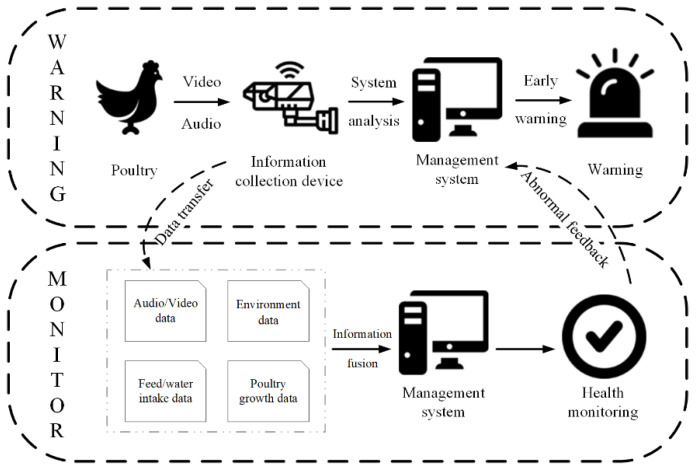
The principle diagram of the method of monitoring the health status of poultry and early warning of disease.

The existing research foundation can be used to realize the detection function of poultry disease. On this basis, combined with the related work of this management system (poultry house environmental monitoring, poultry growth information monitoring, and diet and water consumption monitoring), it can further realize the function of monitoring the health status of poultry. The principle diagram of the method of monitoring the health status of poultry and early warning of disease is shown in Figure 11.

**Figure 12:**
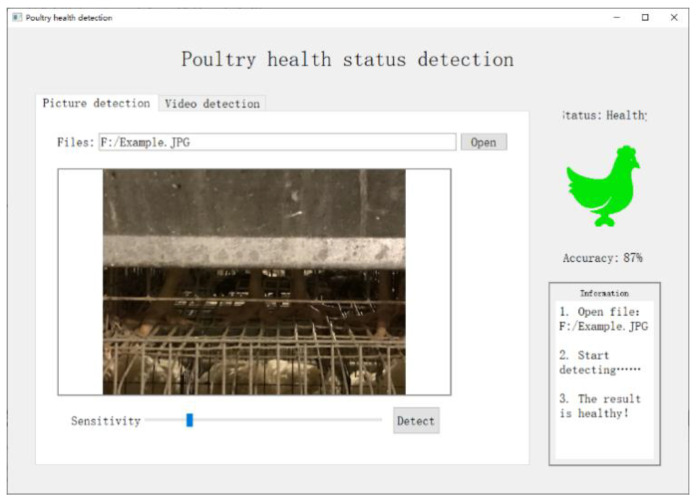
The software interface implemented by the disease detection function; by detecting the visual part of the poultry (the standing area below the feed trough), the current behavioral state of the poultry (standing, lying on the stomach) can be judged, and the health state of the chicken can be inferred.

The software interface implemented by the disease detection function is shown in Figure 12. The poultry pictures and video content are used to monitor the health of the poultry. The detection principle and method are mainly to extract the features of the area to be detected under the food trough in the image, and they use the deep learning method to analyze and calculate the behavior of the chickens in the feature area and then judge the health of the chickens.

## 5. System Performance Test

### 5.1. Wireless Sensor Network Testing

**Figure 13:**
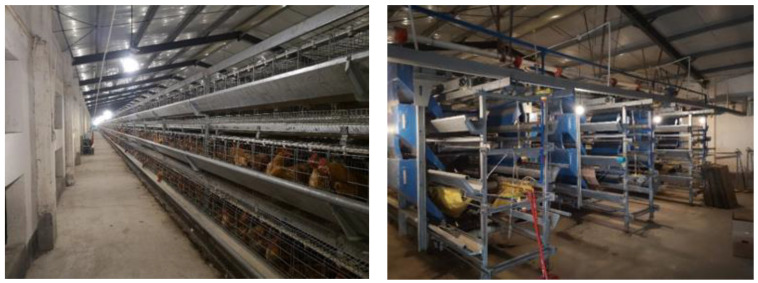
The poultry house of the signal strength and transmission rate test.

The system prototype constructed by this research is deployed in the breeder farm of South China Agricultural University. The test of the wireless sensor network system is mainly carried out on the farm. The on-site environment of the poultry house is shown in Figure 13. There are three rows of chicken cages, and each row is divided into three layers.

In order to analyze the performance of the constructed wireless sensor network in the poultry house, we tested the signal strength, transmission rate, and transmission stability of the environmental information sensing unit.

**Figure 14:**
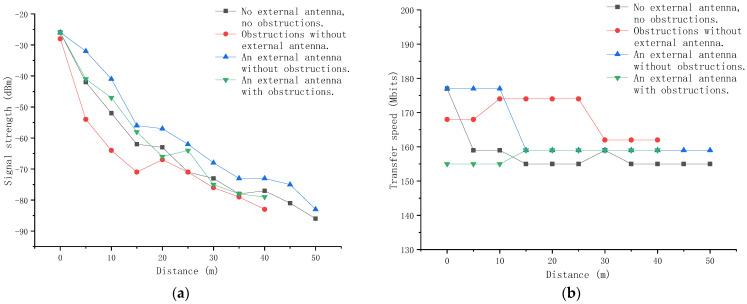
The test results of the environmental information sensing unit: (**a**) signal strength; (**b**) transmission rate.

Figure 14 shows the test results of the signal strength and the transmission rate of the environmental information sensing unit. We tested the signal strength and transmission rate of the environmental information sensing unit under four conditions: (1) No external antennas, cages, or other obstacles; (2) No external antennas, but cages and other obstacles; (3) Equipped with an external antenna, which is not blocked by obstacles such as cages; and (4) Equipped with an external antenna, and is blocked by obstacles such as cages. Every condition was tested three times, and we took the average value as the signal strength and transmission rate after the signal stabilizes for about 30 s.

The test results show that the signal strength of the environmental information sensing unit will decrease as the distance increases, and obstacles such as cages in the poultry house will also weakly affect the signal strength of the environmental information sensing unit; when using a 2 dBi gain antenna, it can effectively improve the signal strength of the environmental information sensing unit, but the transmission speed of the environmental information sensing unit is basically not affected by the distance, and its transmission speed has been stable at 150–180 Mbits. This shows that the environmental information sensing unit is equipped with a 2 dBi gain antenna, which can work normally in a poultry house with a radius of 40 m.

### 5.2. Cloud Database Testing

In this study, the cloud database system used Alibaba Cloud Service (RDS version of cloud database), the database version was MySQL 5.7, the storage engine used was InnoDB, the database memory was 1024 MB, the storage space was 20 GB, and the maximum number of supported connections was 2000. During the test, the performance of the cloud database was monitored for a period of time (one hour). During this period, there were four environmental information sensing units in the poultry house, two poultry house servers for continuous data uploading, and eight users who use the host computer software to read and write the database.

According to the test results of the cloud database performance parameters, the data query task demand is greater than the data upload task. However, the overall performance of the database is stable, the CPU and memory utilization rates are kept at a low level, and the cloud database system runs without pressure.

**Figure 15:**
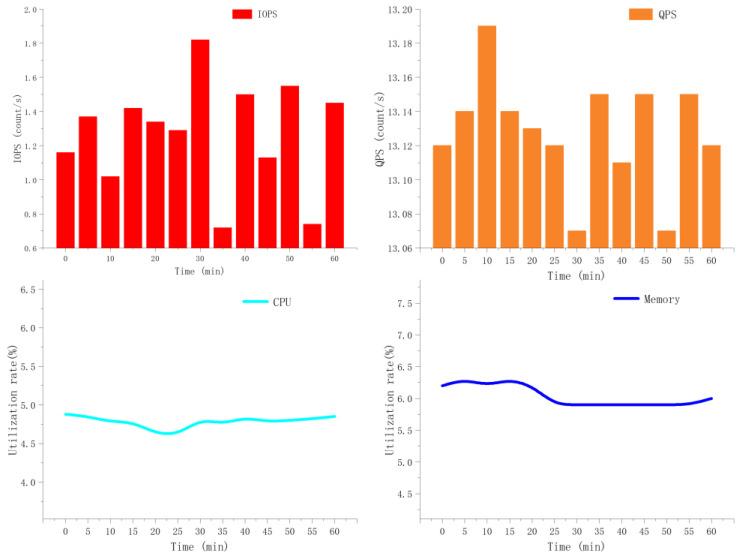
Cloud database performance parameters within one hour. IOPS means input and output operations per second, QPS means queries per second, and CPU means central processing unit.

Figure 15 provides a more intuitive understanding of the operating status of the cloud database. During this period of time, the number of input/output operations per second (IOPS) of the cloud database was basically maintained at about 1.5, and the number of queries per second (QPS) remained above 13, but the utilization of the central processing unit (CPU) and memory has been maintained at a relatively low level (CPU utilization rate is 4.6–4.9%, memory utilization rate is 5.9–6.3%). It can be seen that the operation of the cloud database is stress-free under the experimental conditions, and the normal use of the system can be guaranteed.

## 6. Discussion

Designing a comprehensive and practical poultry farming information management system requires research on three aspects: (1) hardware design and networking of the underlying device, (2) database design and communication related issues, and (3) upper client software design.

LANs and servers should be deployed in poultry houses, and the use of wireless sensor networks (WSN) leads to low-cost and low-power deployments, making them the dominant choice [25]. The sensor is communicated as a child node in the local area network, and the data are connected to the external network through the server to upload the data to the cloud database for storage and query. The data format, transmission protocol, packet loss rate during transmission, and transmission strategy when network failure occurs should be further studied.

Environmental monitoring in poultry houses is the top priority to address animal welfare [26,27]. However, we have only studied a small part of the monitoring work of environmental parameters; there are still many important environmental parameters such as ammonia concentration, dust, and microorganisms monitoring work that need to be further studied. In addition, due to the uncertainty of hydrogen sulfide concentrations in the poultry house, we can consider using more sensitive hydrogen sulfide sensors [28].

How to effectively manage the massive information generated in the system is the primary goal of the future development [11,29]. Through good monitoring and proper storage of data, data mining technology can be used to diagnose poultry disease and provide early warning opportunities [30]. Moreover, using the cloud database as the core hub in this study could also help with the optimization of farming environment and breeding methods.

In order to meet the efficient management and the welfare of animal farming of a different scale poultry farm, this paper establishes a poultry farming information management system based on the cloud database, whose real-time monitoring of environmental information, poultry behavior information, and dietary information in poultry houses are integrated into the system. If successful, the system may meet the business needs of the poultry industry in regard to the environmental monitoring of poultry houses, monitoring of individual growth information, disease monitoring and early warning, traceability, and daily enterprise office management; at the same time, the data information generated during the production process will be well managed, and the poultry farming process informationized and intelligent [31].

## 7. Conclusions

The work reported in this paper builds a poultry farming information management system that covers the information management functions of production aquaculture, corporate office, product traceability, and poultry disease detection, and the unified construction of the system is realized. The system is divided into four modules according to the daily production management needs of farmers. By using intelligent sensors, building a wireless sensor network, and using a cloud database for data storage, a good interaction between modules, software, and hardware is realized, which can bring the animals closer to the farmer. At the same time, the system stores the collected data information in the cloud, and the cloud-based information management system will lead the development direction of the poultry farming management system.

## Figures and Tables

**Table 1:** Comparison of three commonly wireless transmission methods (Bluetooth, Wi-Fi, ZigBee) within four parameters (frequency band, transmission distance, power dissipation, transmission rate).

Transmission Modes	Frequency Band	Transmission Distance	Power Dissipation	Transmission Rate
Bluetooth	2.4 GHz	2–30 m	20 mA	1 Mbps
Wi-Fi	2.4 GHz	100–300 m	10–50 mA	600 Mbps
ZigBee	2.4 GHz	50–300 m	5 mA	100 Kbps

**Table 2:** Industrial control computer performance parameters.

Device	Parameter	Manufacturer
CPU	Intel Core i5-7440HQ @ 2.80 GHz	Intel
RAM	8 GB (DDR4 2666 MHz)	SAMSUNG
Operating system	Windows 10 Professional 64-bit	Microsoft
Hard disk	NT-128 (128 GB/SSD)	Kingspec
Network card	43224AG 802.11 n Wi-Fi Adapter	Broadcom Corporation

**Table 3:** The environmental sensors performance parameters.

Type	Range	Resolution	Accuracy	Model
Temperature/°C	−40~125	0.01	±0.3 °C	SHT20
Relative humidity/%	0~100	0.01	±3%	
Light intensity/lx	0~65,535	0.01	±20%	BH1750FVI
H_2_S concentration/ppm	1~200	0.1	±3%	MQ-136

**Table 4:** Environmental information packet format.

Number	Identifier	Data (Hex)	Size (Byte)	Description
1	EI	45 49	2	Packet header
2	PL	-	4	Packet length
3	UN	55 4E	2	EISU number
4	TS	54 53	2	Temperature data start flag
5	TD	-	4	Temperature data
6	TE	54 45	2	Temperature data end flag
7	HS	48 53	2	Humidity data start flag
8	HD	-	4	Humidity data
9	HE	48 45	2	Humidity data end flag
10	BS	42 53	2	Light intensity data start flag
11	BD	-	4	Light intensity data
12	BE	42 45	2	Light intensity data end flag
13	SS	53 53	2	H_2_S concentration data start flag
14	SD	-	4	H_2_S concentration data
15	SE	53 45	2	H_2_S concentration data end flag
16	CRC	-	4	Check code
17	EOP	FF 45	2	End of packet flag

## Data Availability

The data presented in this study are available on request from the 1st Author (Haikun Zheng).
